# Making the Most of Mealtimes (M3): protocol of a multi-centre cross-sectional study of food intake and its determinants in older adults living in long term care homes

**DOI:** 10.1186/s12877-016-0401-4

**Published:** 2017-01-13

**Authors:** Heather H. Keller, Natalie Carrier, Susan Slaughter, Christina Lengyel, Catriona M. Steele, Lisa Duizer, K. Steve Brown, Habib Chaudhury, Minn N. Yoon, Alison M. Duncan, Veronique M. Boscart, George Heckman, Lita Villalon

**Affiliations:** 1 Schlegel-University of Waterloo Research Institute for Aging, 250 Laurelwood Drive, Waterloo, ON N2J 0E2 Canada; 2 École des sciences des aliments, de nutrition et d’études familiales, Faculté des sciences de la santé et des services communautaires, Université de Moncton, Moncton, NB E1A 3E9 Canada; 3 Faculty of Nursing, University of Alberta, Edmonton, T6G 1C9 AB Canada; 4 Faculty of Agricultural & Food, Sciences, University of Manitoba, 405 Human Ecology Building, Winnipeg, MB R3T 2N2 Canada; 5 Toronto Rehabilitation Institute, University Health Network, 550 University Avenue, #12-101, Toronto, M5G 2A2 ON Canada; 6 Rehabilitation Sciences Institute, Faculty of Medicine, University of Toronto, Toronto, M5G 1V7 Canada; 7 Department of Food Science, University of Guelph, Guelph, ON N1G 2W1 Canada; 8 Department of Statistics and Actuarial Science, University of Waterloo, Waterloo, ON N2L 3G1 Canada; 9 Department of Gerontology, Simon Fraser University, 2800-515 W. Hastings St, Vancouver, BC V6B 5K3 Canada; 10 School of Dentistry, University of Alberta, 5-575, Edmonton Clinic Health Academy, 11405-87 Avenue, Edmonton, AB T6G 1C9 Canada; 11 Department of Human Health and Nutritional Sciences, University of Guelph, Guelph, ON N1G 2W1 Canada; 12 Conestoga College, School of Health Sciences and Community Services, Kitchener, ON N2G 4M4 Canada

**Keywords:** Food intake, Determinants, Dietary reference intake, Long term care homes

## Abstract

**Background:**

Older adults living in long term care (LTC) homes are nutritionally vulnerable, often consuming insufficient energy, macro- and micronutrients to sustain their health and function. Multiple factors are proposed to influence food intake, yet our understanding of these diverse factors and their interactions are limited. The purpose of this paper is to fully describe the protocol used to examine determinants of food and fluid intake among older adults participating in the Making the Most of Mealtimes (M3) study.

**Methods:**

A conceptual framework that considers multi-level influences on mealtime experience, meal quality and meal access was used to design this multi-site cross-sectional study. Data were collected from 639 participants residing in 32 LTC homes in four Canadian provinces by trained researchers. Food intake was assessed with three-days of weighed food intake (main plate items), as well as estimations of side dishes, beverages and snacks and compared to the Dietary Reference Intake. Resident-level measures included: nutritional status, nutritional risk; disease conditions, medication, and diet prescriptions; oral health exam, signs of swallowing difficulty and olfactory ability; observed eating behaviours, type and number of staff assisting with eating; and food and foodservice satisfaction. Function, cognition, depression and pain were assessed using interRAI LTCF with selected items completed by researchers with care staff. Care staff completed a standardized person-directed care questionnaire. Researchers assessed dining rooms for physical and psychosocial aspects that could influence food intake. Management from each site completed a questionnaire that described the home, menu development, food production, out-sourcing of food, staffing levels, and staff training. Hierarchical regression models, accounting for clustering within province, home and dining room will be used to determine factors independently associated with energy and protein intake, as proxies for intake. Proportions of residents at risk of inadequate diets will also be determined.

**Discussion:**

This rigorous and comprehensive data collection in a large and diverse sample will provide, for the first time, the opportunity to consider important modifiable factors associated with poor food intake of residents in LTC. Identification of factors that are independently associated with food intake will help to develop effective interventions that support food intake.

**Trial Registration:**

ClinicalTrials.gov ID: NCT02800291, retrospectively registered June 7, 2016.

## Background

Long term care (LTC), also referred to as care or nursing homes, is a residential option that provides for the instrumental and basic activities of daily living of their clients. Older adults > 65 years of age) are the typical resident and dementia is a common condition that is managed in this setting. Poor food and fluid intake is the primary cause for long term care (LTC) malnutrition [1] resulting in falls, poor function, depression, mortality, poor wound care and decreased quality of life for residents [1–7]. Resident energy intake has been reported to be 1500 kcal/day or less [1, 8, 9] and up to 70% have lower than recommended intakes of protein, fibre, calcium, magnesium, zinc, and vitamins E, C, B6, thiamine, riboflavin, niacin and/or folate [1, 8, 9]. Improving food intake for residents in LTC has been identified as a priority by researchers, decision makers and clients (i.e., residents, families) to promote health and quality of life [10]. Yet, rigorous collection of dietary intake data across a diverse and large sample to truly understand prevalence of poor food intake in LTC is limited. For example, some researchers have used a single day of recalled (by resident or care staff) food intake to represent consumption patterns, resulting in potentially flawed conclusions on the adequacy of food intake [11–13]. Smaller studies with weighed food records, the gold standard, have demonstrated large inter-individual differences [12], but are commonly limited in measurement of covariates and the ability to model these due to the small sample size [12, 14]. Additionally, detailed descriptions of the food intake profile are often missing [15]. Understanding the extent of poor food intake and which nutrients are poorly consumed, is relevant in identifying strategies to support intake for residents in LTC. For example, if vitamin D is low, supplementation may be the best strategy, whereas if energy and a wide variety of micronutrients are low, interventions that increase food intake, such as quality eating assistance, may be required.

LTC malnutrition is both *preventable* and treatable; [1, 16] successful interventions can improve the health and function of residents [9, 17–19]. Innovative, multi-level (i.e., targeting residents, staff, dining environment) interventions are needed to address the problem of inadequate food intake and consequent malnutrition in LTC, as causes are likely to interact [20]. To develop interventions, a good understanding of the problem and its determinants is needed. At this point, we have a *limited* understanding of the problem of inadequate food intake in LTC worldwide, and analyses have often been focused on determinants that cannot be changed [21], such as dementia. However, persons with dementia often require some level of eating assistance [21] and this determinant can be modified; staff can be trained on eating assisting techniques [22], and sufficient time and accommodations can be provided to promote eating independently (e.g., finger foods) [23]. Thus investigations focused on describing and determining the relative importance of these amenable factors is needed to make advancements in improving food intake and thus malnutrition in LTC.

Research to date has failed to use a comprehensive conceptual framework to understand and intervene on the multi-level and inter-related determinants of food intake in LTC residents [7, 15, 20, 24]. The proposed study is built upon the M3 concept [25], which has its origins in the *Social Ecological Model* [26], the *Five Aspects of Meal Model* [27]*,* and the *Mealtimes as ‘Active Processes’* substantive theory [28]. The M3 conceptual model suggests that multi-level determinants influence food and fluid intake of residents. Specifically, it is hypothesized that regional government regulations and standards, LTC home policies and features, staffing levels, knowledge and practices, and resident characteristics will influence food intake. Within each of these levels, factors in three domains of *Meal Quality* (nutritious, appealing food); *Meal Access* (oral health, swallowing problems, eating ability); and *Mealtime Experience* (dining environment) are relevant to food intake. This model drives the design, data collection, analysis and interpretation of results in this project [25].

Meal quality is operationally defined as food and fluid offerings that are preferred and culturally appropriate, appealing (smell, taste, appearance), served at an acceptable temperature, and meeting the nutritional requirements (i.e., Dietary Reference Intake [DRI]) of residents. Quality food is favoured over oral nutritional supplements or meal replacements by residents and family for meeting nutritional needs and enhancing quality of life [29]. Government policies and home-level practices with respect to menu planning, choice of commercial or in-house food products, food variety, food budget, and the mandated role and time allocated to clinical dietitians and other allied health professionals (e.g., occupational therapists and speech-language pathologists) all have the potential to influence the types and quality of food provided in LTC, yet we know little about these determinants. Evidence to date suggests that increased funding for food improves energy intake in LTC residents [15]. A shorter menu cycle length is also associated with increased malnutrition [30]. It has been noted that LTC menus have been found to be low in protein and micronutrients [1, 31] and are likely insufficient to support adequate health and function [32]. Of specific concern are modified-texture foods (e.g., pureed) that are frequently provided to persons with dysphagia (i.e., swallowing impairment) to manage their swallowing difficulty. These foods often have poor sensory appeal and low nutrient density [33, 34].

Meal access is operationalized as those factors that influence food/fluid access and specifically: the availability of food (meal timing, between meal snacks); the ability to taste and smell; dysphagia; dentition and oral health status; capacity with self feeding, time it takes to eat, and any eating challenges; and the time provided for and quality of eating assistance. Taste and smell are commonly impaired in older adults and specifically in those with dementia [35, 36], potentially impairing food intake and nutritional status [36]. Dysphagia is a significant comorbidity that influences food intake and malnutrition [21], although prevalence in LTC is elusive. Additionally, dental factors such as loose teeth, poorly fitting dentures, and/or poor oral health may contribute to limited food intake, preference for foods low in micronutrients but easier to eat (e.g., ice cream, commercial puddings, mashed potatoes) or prescription of modified-texture foods (e.g., minced meat) or diets [37]. Oral health status of LTC populations is rarely investigated, but problems are prevalent with estimates of 37% of residents reporting a dry mouth and 51% having untreated cavities [38]. The resulting pain and distress of poor oral health has been shown to affect food intake and malnutrition [21, 37]. In addition, difficulty accessing food due to packaging, lids, and dishes causes stress at mealtimes and is associated with poor food intake [30, 39]. Agitation and decreased ability to eat independently also result in decreased consumption [40]. Requiring assistance commonly results in inadequate food intake [7, 21, 39]. Assistance with food intake ranges from setting up the meal and opening packages, to encouragement, to partial assistance with some foods or total assistance for those unable to eat independently. For those requiring assistance, food access is influenced by the number of qualified, trained care staff, the number of residents requiring assistance, the type of assistance needed, and the presence of family, volunteers or paid meal helpers who can provide one-on-one assistance [22]. For instance, a staffing ratio of 3:1 (residents:staff) as compared to 5:1 significantly improves energy intake for residents in LTC settings [15]. It is anticipated that as many as 50% of LTC residents requiring total assistance could consume at least some of their food independently if changes were made to the environment and supports were put in place to promote resident autonomy [23, 41], such as sufficient time to eat [22].

Mealtime experience is operationalized as the physical and psychosocial mealtime environment and mealtime processes that can influence food intake [42]. Mealtimes can be the highlight of the day for residents, providing opportunities for social interaction as well as development of relationships with care providers and tablemates [43]. Apathy and depression have been found to be independently associated with weight loss in LTC residents [44] and may be influenced by a negative mealtime experience [42]. Two theories specific to persons with dementia (*Mealtimes as Active Processes in LTC* and the *Life Nourishment Theory*), demonstrate the importance and potential influence of the psychosocial environment on food intake [28, 45]. It is hypothesized that positive social connections and honouring individual identities (e.g., food preferences) at mealtimes will promote food intake and quality of life. Consistent with these theories, family style dining provides greater opportunity for social interaction and choice of food offerings and has been shown to improve energy intake in LTC [46–48]. The Eden Alternative®, which is a ‘household’ model providing care to a small number of residents in a resident-centred and homelike setting has some benefit with respect to maintaining body weight [49]. This association may be linked to tailored individualization of care including participation in food preparation, meal choices and honoring food preferences. Social models of care that recognize and encourage staff-resident relationships and resident-centered care are seen as the preferred approach to promote residents quality of life in LTC [49, 50]. To date, the relational and resident-centeredness of mealtimes has been qualitatively explored [43, 47, 51, 52], but has yet to be quantitatively assessed to determine its association with food intake. The physical environment can also influence eating [41, 53] and ‘homelike’ environments with music, decorations, and table dressings have been shown to improve residents’ food intake [46, 54]. Food being plated on the unit, rather than in the kitchen, enhances consumption [54]; residents see and smell the food and can interact with staff about choosing their meal and desired portions at the point of service. On the other hand, bulk delivery systems can create noise and distraction [39].

In sum, we lack rigorously measured dietary consumption data to understand the prevalence of inadequate food intake in LTC residents. Multi-level determinants that address meal quality, meal access and mealtime experience have yet to be collectively assessed to understand their relevance to food intake. Without this knowledge, we cannot undertake the development of effective interventions and policy directives to address the high prevalence of LTC malnutrition and its costly human and system consequences. Specifically, determinants amenable to an intervention, which could have strong potential to improve food intake, have rarely been explored. The objectives of this cross-sectional, multi-site study were to:

1. establish the prevalence of inadequate energy, protein, micronutrient and fluid intake of residents in LTC, across and within four Canadian provinces, and;
2. identify the independent and inter-related associations between multi-level (i.e., resident, unit, LTC home, province) determinants of energy and protein intake of residents in Canadian LTC homes.

The purpose of this paper is to fully describe the protocol used to rigorously measure food intake and to examine determinants of food and fluid intake among older adults participating in the Making the Most of Mealtimes (M3) study.

## Methods/design

**Fig. 1:**
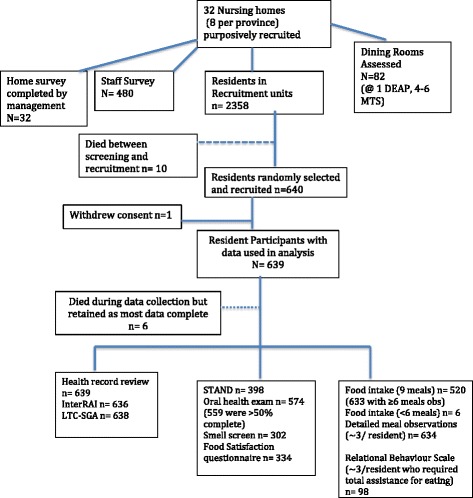
Overview of Data Collection

M3 is a cross-sectional, multi-site study with data collection in four provinces in Canada: Alberta, Manitoba, Ontario and New Brunswick. Regional variations in policy and funding exist within the LTC sector in Canada [55, 56], and thus inclusion of diverse regions supported obtaining a more comprehensive understanding of determinants of food intake. Homes within each province were purposively sampled to also attain diversity with respect to size, model of care, profit-status, cultural factors, rural/urban region and other home-level determinants that could impact food intake [15]. Sample size estimation was based on both objectives, and considered the effect of provincial and home clusters and the intra-class correlation (ICC) between residents within the same facility. Based on previously collected energy intake data from two Canadian studies involving several homes, the ICC (ICC 0.17 and 0.04, with averages of 1511 and 1180 kcals, respectively) [8, 57] was used to determine that a sample of 20 residents in each of 32 homes would provide sufficient sample size to estimate energy intake with a 95% confidence interval of plus or minus 56–58 kcals for the entire sample, and plus or minus 112–116 kcals for provincial samples. Hierarchical multiple linear regression will be used to identify the independent determinants of energy and protein intake of residents. Sample size in regression modeling is dependent on many factors including covariate measurement error [58]. To attain our final sample size we recognized that multi-level models involving covariates at the LTC home level require an increased number of homes rather than an increased number of participants within homes. Previous LTC research using multi-level regression analyses with food intake determinants found 714 residents sufficient for multivariate modeling across levels (LTC home, resident) [15]. Based on funding constraints, the final sample recruited for this data collection was 20 residents per home, from eight homes in each province (*n* = 32 homes), for a final sample of 639 residents (1 participant withdrew consent). Data were collected at the resident, dining room, care staff and home level for this study. Researchers were in the home for an approximately 1-month period to collect all data. Figure 1 provides an overview of the sample and flow of data collection.

### Participants

Eligible LTC homes had been in operation for at least 6 months; had a minimum of 50 residents meeting resident eligibility criteria as described below; and had unique characteristics that promoted home diversity in the sample (e.g., rural, cultural emphasis, size, continuum of care), including profit structure (for profit *n* = 10, not for profit homes *n* = 22). Within each LTC home, we recruited residents from one to three randomly selected care units (depending on size) where a care unit was defined as: 1) a geographic area in a LTC home, 2) having a consistent, assigned group of direct care staff and 3) its own dining area, if possible. One of the selected units per home was a dementia specific unit, if such a designation existed for the home, to ensure the inclusion of persons with dementia in the sample. All residents within these units, regardless of cognitive ability, were eligible to participate if they were: over the age of 65 years (one participant was 62 years); required at least 2 h per day of care due to dependence in activities of daily living (e.g., bathing); resided in the home for at least 1 month; and, either they or their substitute decision maker provided consent to participate. Residents were excluded if they were currently medically unstable; were a temporary resident in LTC (i.e., respite or convalescent care); required tube feeding; were not eating anymore because they were at the end of their life; did not routinely eat in the dining room; or had advanced directives excluding them from research participation. Care staff was trained on these criteria and identified those residents who were eligible [8]. Eligible residents from each unit were collated and a random number table was used to identify the order in which residents should be approached by the trained care staff member to determine if they would be interested in hearing more about the study from the researchers. An alternative decision maker (ADM) was approached for residents who had a Cognitive Performance Scale (CPS) from the Minimum Data Set that indicated they had moderate/high cognitive impairment (i.e., 3+) or where the care staff believed the ADM should also be approached. Training and standardized scripts were provided to the care staff to support recruitment. To determine representativeness of the participants for their units, age, gender and cognition (CPS) were noted for all eligible residents. Care staff was eligible to participate if they were employed by the home (fulltime/part time position), they were direct care staff (e.g., nursing, recreation, dietary aids) and if they worked on the selected study units on the days of this data collection.

### Ethics approval and consent to participate

Ethics approval for the study was obtained from the Research Ethics Boards of the Universities of Alberta, Manitoba, Moncton, Waterloo and the University Health Network, Toronto. Some LTC homes had an individual review committe, and if so, ethics approval was sought. Informed written consent was obtained by the researchers directly from residents who had the capacity to consent, or from their ADM. Many of the initial recruitment meetings between the researcher and ADMs were conducted over the telephone. The researcher described the study procedures in detail verbally and if the ADM was comfortable with this information, they had the option of providing verbal consent immediately. This was followed with the mailing/emailing of the information letter and consent form to the ADM and having the written consent returned by mail or email. Residents who were able to provide their own consent did this in writing at the time of recruitment. During data collection, we continually evaluated assent to participate in the study by the willingness of residents to cooperate with various data collection procedures [59]. Care staff provided informed consent to complete their questionnaire and the home signed a research agreement noting their cooperation with all aspects of the study including completion of a home survey.

### Measures and data collection

Data were collected between January and December 2015 in each of the 4 provinces. Provincial coordinators were extensively trained face-to-face on all procedures; three of four of these researchers were registered dietitians, while the fourth had been trained in dietetics and had extensive applied nutrition research experience. These coordinators subsequently trained two research assistants (RAs; post graduates, typically health students) per province to collect food intake and meal observation data for the resident participants. All of the data was collected either at the home, dining room, staff or resident level.

### Home/staff level measures

A home-level questionnaire was used to collect variables influenced by provincial policy (e.g., food budget allocation, dietitian clinical time) and home-level policy and practices (e.g., staff training, menu planning process) and characteristics (e.g., age of home, size, type of food production). This questionnaire was provided to the director of care and the food services manager at the beginning of the study. In addition, the food service manager was asked to provide the full menu for nutrient analysis and an assessment of variety (first week of the planned menu, regular and pureed first choice; variety only for regular menu). Home recipes and specific food products (including manufacturer nutrient analysis), as well as standard portion sizes for estimated foods, was used to complete an accurate nutrient analysis. This analysis was completed on site by the RAs in the event that they needed to direct questions to the food service manager on menu specifics (e.g., type of margarine, meal substitutions). Table 1 provides further detail about the home questionnaire.

**Table 1:** Home-level variables collected in the Making the Most of Mealtimes Prevalence Study

Home Level Characteristics		
Staff perceptions of Person Directed Care (50 items)	Food production system	How food is purchased (e.g., purchasing group)
Profit/non-profit status	Proportion of food commercially produced	Monthly food cost per resident and if included oral nutritional supplements
Home provides various care levels in one setting eg. Independent living, retirement, long term care	How modified textures produced or if purchased	Cost of oral nutritional supplements per month
Age of home	Production of thickened fluids	Vitamin/mineral supplements included in raw food cost and approximate cost per month
Types of care provided in home and number of beds	Timing of meals/snacks	Weighing procedures for residents, type of scales available
Staffing levels (nursing, dietary aids/food service workers, cooks)	Multiple seatings for meals to accommodate all residents	Training of food service staff
Specialized staffing levels (chef, director of food service, clinical dietitian, speech language pathologist, occupational therapist, recreational therapist)	Main meal of the day	Food safety monitoring and training of staff
Access to specialized services (e.g., dentist/dental hygienist)	Availability of food and fluid outside of meals	Quality improvement initiatives in the past year
Who involved in menu planning (including residents/families)	How special occasions observed at meals	In-service training for food service staff
Latest revision of menu	How seating arrangements at meals determined	Policies and procedures for sickness with food service employees
Standards for menu planning	Ability to store food in resident’s room or common fridge	How need for modified textures determined
If and how the menu is seasonally changed to accommodate available foods	Ability to order in restaurant food from outside of the home	Availability of food/nutrition support services
How resident food choice determined	Ability to have meals delivered to their room (and if additional cost required)	Areas of improvement in nutrition, dining, meal service desired by the home and barriers to accomplishing
Food delivery system	Eat with family in the home	Diet (e.g., diabetic) options available

A minimum of 10 care staff on the study units completed the valid and reliable Person-Directed Care measure [60, 61], which collects staff perception of resident-centered care and organizational support with 50 items. Demographic information was also provided. To reimburse their time a $5 gift card for a national coffee shop was provided upon completion. Although we had planned to have care staff complete this data collection on laptops, almost all staff preferred a paper and pencil version of the questionnaire.

At the unit level, the physical and psychosocial dining environment was assessed. The Dining Environment Assessment Protocol (DEAP) is a face valid and inter-observer reliable instrument that assesses key design features and rates the dining space on homelikeness and functionality with summative scales (1 = low, 8 = high) [62]. The DEAP was completed once by the provincial coordinators, when the dining areas were empty, for each of the 82 dining areas included in the study across the 32 homes. A second measure, the face valid and inter-observer reliable Mealtime Scan (MTS) [63], was completed 4–6 times over the duration of data collection in a home, by coordinators in each dining space when occupied, with all meals (breakfast, lunch and supper) represented at least once. Number of persons, ambiance (e.g., table dressings), physical environment (light, temperature, and noise with an environmental meter (Shimana SE-DT-8820) using a standardized protocol) [64] and Mealtime Relational Care checklist (M-RCC) for care staff mealtime practices were observed and tallied/scored. This latter scale focused on care staff practices that were dignified (e.g., using napkin to wipe mouth), supported resident participation in the meal (e.g., clearing plates), promoted social interaction among residents and care staff, and attended to key hospitality concepts (e.g., not talking over residents to other staff, clearing tables promptly); positive and their contrasting more negative behaviours were tracked. Three summative scales (1 = low, 8 = high) on the physical environment (e.g., light, sound, temperature), social, and person-centredness of the entire dining environment were completed at the end of each meal after all observations on the MTS were made; individual variables/sections on the MTS were used by coordinators to rate these aspects of the meal. The ratings for the 4–6 MTS observations conducted in each dining room over the duration of the study in a home were summed to determine an average for the dining room. Table 2 provides further detail on these measures.

**Table 2:** Dining room variables collected in the Making the Most of Mealtimes Prevalence Study

Dining Environment Assessment Protocol (DEAP) [62]		
Type of unit (e.g., segregated dementia)	Diagram of physical layout	Rating of lighting intensity and glare
General physical space e.g., # chairs, tables, staff stools, entryways, garden/outside views	Components that promote safety e.g., contrast, rounded edges of furniture, adjustable tables, secured toxic substances, short distance from bedrooms	Components that are homelike/promote orientation e.g., dining room open between meals, clock, accessible kitchen for residents/families, television, posted menu
Residents opinions on noise, light, temperature responded to	Size of space rated on homelikeness	Pathways rated on length for meal delivery, and safety
Presence of obstacles/clutter in dining area	Functionality of space so that staff can view all residents	Use of restraints (lap, chair)
Mix of seating arrangements (e.g., 2, 4, etc. tablemates)	Overall rating on homelikeness (1 = low, 8 = high)	Overall rating on functionality of space (1 = low, 8 = high)
Mealtime Scan [63]		
Temperature, luminescence, humidity, sound (@ 4 ×/meal)	Number and types of persons in dining room and adjacent areas if eating	Number eating in own room
Any food production/delivery issues (e.g., change in menu, problem with food, short staffed)	Orientation cues e.g., food odour, clock, table decorations, table cloth/settings, contrast	Residents involved in mealtime activities
Noise that could be distracting and source e.g., crushing medications, hallway chatter etc.	Television, music (source and type, loudness level obstructed conversation)	Mealtime-Relational Care Checklist (*n* = 26 positive and negative items)
Overall rating of functionality of physical space (low = 1, high = 8)	Overall assessment of social space (low = 1, high = 8)	Overall assessment of person centred care (low = 1, high = 8)

### Resident level measures

Extensive data at the resident level were collected for health, nutritional status and potential risk factors for poor food intake. Only key aspects are presented in the text as Table 3 outlines measures in detail. For almost all residents, three nonconsecutive food assessment days, including a weekend day, were used to determine food intake of participants; for a few residents, logistics required consecutive days being observed. Each food item on the main plates was weighed before and after the meal to determine the amount consumed by subtraction and food wastage due to spillage was estimated, where possible, for the unconsumed portion [65]. Fluids and side dishes were estimated, with the amount determined from the detailed production menu, as well as by measuring vessel/cup size prior to data collection. Two RAs completed this data collection on five residents per meal per unit. If a resident was not present on the unit for the meal, an alternative day was chosen to promote as complete a data collection as possible. RAs also estimated food and fluid intake for the participating residents at snack times and between meals, identifying source (i.e., food brought in by family/resident/staff) and consumption by observing and/or asking the residents, family and/or staff. Care staff on the unit were asked to report any additional food intake such as before-breakfast food consumption, and were trained to record the consumption of evening snacks and beverages on the food intake assessment day. Other mealtime behaviours (e.g., time to eat, number of assistants helping the participant etc.) were recorded at each meal.

**Table 3:** Resident-level variables collected in the Making the Most of Mealtimes Prevalence Study

Resident Data Collection		
Date of birth, gender, ethnicity, months since admission	Food brought in/purchased by resident/family	Resident Food and Foodservice Satisfaction Survey [79]
Weight history for past 6 months	Family provides micronutrient supplements	Smell screening with Sniffin Sticks (*n* = 12 odours) [78]
Diet/fluid texture prescription	Mini-Nutritional Assessment- Short Form (screen for nutritional risk) [70]	Relational Behaviour Scale [67]
Other therapeutic diet prescription	Knee height (to estimate standing height)	Weighed food intake, main plate; estimated side dishes/beverages
Cultural meal preferences met	Ulna length (to estimate standing height) [87]	Between meal snacks/beverages estimated
Use of oral nutritional supplements	Calf circumference	Ed-FED (× 3 meals) [66] and 9 additional eating challenges
Diagnoses (based on InterRAI LTCF categories) [71]	Patient Generated- Subjective Global Assessment to assess malnutrition [69]	Mealtime-Relational Care Checklist (× 3 meals) [63]
Medication (dose, frequency)	STAND (dysphagia screening instrument) [77]	Time in dining room, taken to eat (× 9 meals)
Use of antibiotics, psychotropics, vitamin/minerals	Oral health exam (e.g., teeth count, observance of problems, pain, opinion on need for urgent dental care, oral health affect food intake) [78]	Number of assistants during meals and whom; served or assist with eating (× 9 meals)
Any acute change that could affect food intake	InterRAI LTCF (selected items) [71]	Leaving dining room during meals/wandering

At one meal per food intake assessment day for a resident, a more elaborate mealtime observation was completed. This included the Edinburgh Feeding Questionnaire (Ed-FED) which scores eating challenges and assistance required [66]; 9 additional items that describe further eating challenges (e.g., does the resident get distracted, do they cough during the meal, choke etc.) were also recorded and scaled to be consistent with Ed-FED (never, sometimes, frequent). Two further tools were used to assess the interactions between care staff and the resident at this meal. For those residents requiring total eating assistance to complete a meal, the Relational Behaviour Scale [67] was also completed. The M-RCC checklist from the MTS (described above) was completed on all individual resident participants, regardless of requiring eating assistance, to observe interactions between care staff and the participant. Averages across meals will be used in analyses.

Nutritional status was assessed with Subjective Global Assessment [68]. The Patient-Generated version, originally designed for oncology outpatients [69] provides more landmarks for a physical exam as well as risk factors for poor food intake. As many residents are unable to be an informant, sections typically completed by the patient (e.g., weight change, risk factors for food intake) were gathered from the chart, staff or family members. Source of information was tracked. The Mini- Nutritional Assessment Short Form was also used to determine malnutrition risk [71].

The interRAI LTCF [71] provides a standardized and validated means of collecting comprehensive clinical information on LTC residents and scales for cognition (CPS), pain, activities of daily living, depression (Depression Rating Scale - DRS), and challenging behaviours (Aggressive Behaviour scales - ABS) [72–76] can be derived. Items to complete these scales as well as the oral health/nutrition items were collected by provincial research coordinators who interviewed care staff currently providing care to the resident. Care staff were asked to reflect on the last 3-days for reporting needs and behaviours of the resident.

Dysphagia risk was a composite variable. If the resident was on a thickened fluid diet, they were deemed to be at ‘dyphagia risk’. For all other resident participants, a screening procedure was completed to determine risk for swallowing problems. The provincial coordinator (or in the case of Ontario, a doctoral student who was a speech-language pathologist) completed the standardized Screening Tool for Acute Neuro Dysphagia (STAND) [77]. This brief screening protocol has been validated against a modified barium videofloroscopy and has 92% sensitivity and 60% specificity for detecting aspiration [77]. For this protocol, residents consumed three teaspoons of applesauce and drank 90 ml of water, in a continuous fashion; signs of dysphagia (i.e., coughing, wet voice quality, throat clearing) were noted. If the participant passed the STAND without signs of swallowing difficulty but later displayed coughing or choking during meal observations (as captured through the additional eating challenges questions), they were considered to also be at risk for dysphagia.

Dentition and oral health were determined by a standardized oral assessment based on the Canadian Health Measures Survey [78]. In each province a single contracted and trained dental hygienist experienced with the LTC population completed this standardized oral assessment for the participating residents. For residents with adequate cognition, olfactory ability was assessed using twelve “Sniffin’ Sticks” (Burghart Messtechnik GmbH) [79]. Participants sniffed with each nostril an olfactory ‘pen’ containing a known smell and attempted to identify the odour from a list of four pictures with word labels (e.g., lemon, coffee). Satisfaction with food and foodservice was also completed by these residents using a 21-item interviewer-administered tool, valid and reliable for persons with and without dementia [80]. Residents with mild/moderate dementia were asked to complete these assessments (i.e., CPS 1–3); if the provincial coordinator perceived difficulties with completion (i.e., resident needed multiple prompts to answer the question, forgot the question), the assessment was stopped.

### Data management and analyses

REDCap™ (Vanderbilt University, Version 6.2.2), a data entry/capture system was used to promote data security, and resident and staff data was sent in this electronic format to the study centre at the University of Waterloo. An information specialist was contracted to develop the REDCap™ forms and served as a consultant throughout the project until all data collection was completed and data were cleaned, exported and merged. Paper and pencil copies of forms were used when computer collection was not possible (e.g., meal meal observations, patient assessments, home survey ). Many LTC homes did not have access to WiFi, thus encrypted laptops were used during data collection to promote security. A protocol was followed for routine downloading of data to a secure server at University of Waterloo. Hard copy data were kept in a secured file box and kept in locked storage when not in use. Data were cleaned using an extensive procedure by the lead provincial coordinator and an analyst; data checking was completed where necessary with sites (e.g., home questionnaire). Food Processor™ software (ESHA Research, Version 10.14.1) using the Canadian Nutrient File was used for nutrient analysis of food intake and menus. Recipes from homes were also inputted into the system. The Food Processor files were sent through a secure file transfer process to University of Waterloo for extraction of nutrient information and assembling into the main data set. Home survey, dining room observations and staff questionnaires were inputted into the resident file to ensure that all data were merged at the individual resident case level in the data set.

The data were collected at multiple levels and are considered ‘clustered’ at the province, LTC home and dining room levels. Thus, multivariate models including variables from more than one level (i.e., home, unit, resident) will require nested or hierarchical analysis. Prevalence of inadequacy of micronutrient intakes will be evaluated through comparison of intakes to the Dietary Reference Intake using the Estimated Average Requirement cut-point method [81]. This analysis will take into account the inter- and intra-individual variation inherent in dietary assessment [82]. Analyses will be completed with and without micronutrient supplements. Analyses relating dietary energy and protein intake (outcomes) to potential determinants will be based on the theoretical M3 model. Initial analyses will be conducted by level to screen for important relationships using hierarchical multi-level regression models. These models will contain indicator variables for province, and a random effect for homes and units, as well as the independent variable under consideration. Variables with p < 0.2 will be retained to develop multivariate models. Retained home, dining room, staff and resident-level variables, will be incorporated into the final multi-level models. Collinearity and interactions will be assessed in final models. Backwards elimination will be used to develop the final multi-level models. A variety of descriptive, bivariate, and multivariate analyses are also planned based on key questions posed by the co-investigators to make full use of this exceptional dataset (e.g., Is dysphagia risk associated with time to eat, oral health etc.).

## Discussion

This unique study not only has resulted in a rigorous data collection that will be invaluable for understanding food intake and care with respect to nutrition of residents in LTC, but also provides important insights for researchers on the importance of cluster design and analysis in understanding these questions. The following discussion further elaborates on what has been learned as a result of conducting this study so far.

### Building research relationships with homes and staff

A significant barrier to LTC research is receiving consent from homes, staff and residents to participate in the data collection. The best way to ensure participation is to build a trusting relationship [83]. We recruited 30 of 32 homes purposively at the time of submitting our proposal for funding; each provincial investigator leading the project in their province conducted this recruitment based on their prior relationships with these sites. All of these initially recruited homes participated in the project. Confidentiality of home identity was also assured to promote feelings of trust [83] and a memorandum of understanding between the university and the individual LTC homes was completed. This agreement was reviewed and signed by home management in a face-to-face meeting with the researchers and/or the provincial coordinators in each province approximately two months prior to starting recruitment of participants in that home. Facility management, director of care (nursing), food service management and the clinical dietitian were involved in these initial meetings and all signed this agreement to ensure good communication about the study. A primary contact person for each home was determined and this person was involved in all communications that supported the data collection. In most LTC homes, this role was assigned to the director of food service, but in other homes, it was the clinical dietitian. Post data collection, a summary document described nutrition risk, risk for dysphagia, oral health, and any other concerns for each participant. This summary was provided to the home’s dietitian and primary contact person to support the resolution of these potential problems. As well, most homes were interested in their menu nutrient analysis and this information was provided to them within a month of the data collection. When the data collection was finished across the country, home representatives were invited to a stakeholder meeting, which included government representatives, decision makers/knowledge users and other local LTC homes to present site-specific anonymous data on key variables for the province. This helped to build awareness of the study, to start work with respect to improving provincial policy, and to begin intervention development. In separate individual face-to-face meetings, each home was provided with their specific results, benchmarked with other homes in their province. All homes were receptive to this engagement and expressed appreciation for being involved in the study and receiving their own data to support quality improvement initiatives. As a result of these relationship building efforts, many homes have expressed interest in being part of further research focused on developing and testing interventions to improve food intake of residents.

As the researchers were in the home for approximately 20 days in a month, a clear understanding of their purpose and the expectations for staff around resident data collection were needed. A variety of strategies were used to promote this communication and build positive relationships. Management discussed the project with staff on the study units, using a script provided by the researchers. A similar script was provided for communication books. The provincial coordinator was the first researcher to enter the unit and started with training the seconded home staff to determine resident eligibility. They were typically introduced to key staff members by the home contact person and procedures for reviewing resident charts were discussed. DEAP was conducted on the first or second day by the coordinator as a way of initiating data collection for the units. All researchers wore nametags and were trained to be as unobtrusive as possible. The provincial coordinator was on site the first day that the RAs entered the home; it was found to be beneficial if the RAs could be introduced to key staff members and shown around the home and the study units the day before they began mealtime observations. The researchers worked as a team to identify residents and ensure a complete data collection. However, staff cooperation was needed for help in initially identifying and finding participating residents as they were often not in their rooms and arm bracelets for identification were not typically used. Where staff was needed for resident data collection (e.g., risk factors for poor food intake as reported by care staff for the PG-SGA), care staff were asked when a convenient time would be for this brief data collection. Where more intensive time was required (e.g., interviewing care staff for completion of interRAI LTCF), the home provided an additional staff member for coverage and this cost was billed to the project. All of these activities helped to build a positive relationship with the staff and supported a quality data collection from residents.

Staff participation was promoted by providing back-fill for staffing on selected days when the staff PDC questionnaire was completed. Staff also appreciated the $5 gift certificate. As discussed, most of the staff members were hesitant to use the provided laptops for their data entry and hard copy versions of the questionnaire were used. They required additional assurance that their individual results would remain anonymous and in no way would home management know who participated. When results were presented to management, staffing classification of ‘nursing’ or ‘other’ was only used to promote anonymity of staff that was limited in numbers (e.g., recreation staff).

### Recruiting residents

Homes were chosen to have a sufficient number of residents (~50) to ensure that 20 eligible residents could be recruited. In some sites, random selection of the unit to recruit resident and care staff participants was not possible due to the size of the home. A care staff member, typically a regulated nurse (RPN/LPN), was shown how to: a) develop the list of eligible residents from the randomly selected study units, b) randomly select residents and/or the alternative decision maker (ADM) to be approached using a random number table, c) use a script to speak with the resident/ADM, and d) note reasons for lack of interest in the study. This training took a significant amount of time on the part of the provincial coordinator and specialized forms, tables and written instructions were provided to the home to make it as error-free as possible. The staff member randomly recruited 40 participants, as we had determined from a pilot that approximately 1 in 2 residents/ADM will refuse participation. This took a significant amount of time; the home invoiced the research project for the hours required by the seconded home staff member who completed this work.

Many homes struggled with this first step of data collection and we did not get complete records on participation rate as a result. However this two-step process for attaining consent resulted in only minimal problems with withdrawal post recruitment. Based on records available from homes, 84% of eligible residents/ADM reached by home staff agreed to be contacted by researchers. Not all eligible residents were contacted by the researchers; if they reached the 20 patient quota per home, the remaining list of eligible participants who had agreed to be approached by researchers was not exhausted. As resident participation rates for Canadian LTC research ranges from 65–72% [83–85], M3 had a successful participation rate which is attributed to the relationship building, training of home and research staff, as well as the non-invasive protocol. Participants were representative of the units where they lived. Specifically mean age (86.9 SD 7.81 vs. 87.0 SD 8.2 respectively), and proportions of males (male 32% v. 31.5% respectively) or those requiring an ADM for consent (74.6% vs. 72.8% respectively) did not differ between participants and eligible non-participants. Attrition due to death was minimized by recruiting and collecting all data within one-month. Ten eligible residents died between identification by the home staff (screening) and recruitment by the researchers, while six participants died during data collection. These latter participants were retained however, as they each had a minimum of two days of observed food intake, and thus provided key data for analysis. Where prudent, sensitivity analyses will be used to determine if these patients unduly affect interpretation of findings.

### Data collection

We opted for tools and scales that had a minimal burden for residents to promote a complete data collection and chose where possible tools that were familiar to homes and staff (e.g., interRAI LTCF). As a result, we achieved high completion rates for most resident-level measures: PG-SGA (99.8%), calf circumference (98%), STAND (69.7%; 398/517 not on thickened fluids), oral health exam (89.8%; 574/639, 559 (87.5%) with complete data on exam), food satisfaction (52.3%), and smell test (47.3%). The latter two were expected to be only completed in those residents with sufficient cognition to do so, which limited the sample for these measures. STAND was not completed for residents that consumed thickened fluids (10.6%), as we did not want to expose these residents to the risk of aspiration; the remainder of resident participants where STAND was not completed either refused participation or were unable to follow the simple commands required. The proportion that completed STAND demonstrated the importance of an a priori decision to create a composite variable to identify dysphagia risk, which would ensure 100% identification of this important covariate. Dysphagia risk was defined as: thickened fluid prescription or failed STAND or exhibited any coughing or choking during three observed meals.

As noted, data collection on food intake was rigorous with protocols for weighing and estimating food clearly outlined and followed by the RAs. There were some challenges in attaining non-consecutive days or timing. Specifically, where travel from the provincial research centre required an overnight stay, the nonconsecutive day requirement for meal observation could not always be adhered to. We were able to observe 6+ meals for almost all participants (99%) with only 6% of participants having one or more meals that were not observed as they were consumed outside of the home. With a minimum of two days of intake for all residents, and little missing data for other days, we consider this data sufficiently thorough to address the primary research questions. The poorest collection on food intake was the evening snack as estimated by care staff. Other than using reminders with evening staff as well as clear instructions and daily follow up, no further suggestions can be provided to support complete and accurate data collection when RAs are not in the home to observe food intake. However, as 80–90% of calories are consumed during meals in LTC [86], we anticipate minimal bias in our primary outcomes due to inaccuracy in collection of consumption of food in the evening (after supper) or overnight. Nutrient analysis protocols promoted rigor and consistency. If a home recipe was unavailable, the RAs were trained to use the following steps: 1) use another home’s recipe for a similar food item in their province; if this was unavailable, 2) use a recipe from another province; and if this was not available, 3) consult Food Processor™ standard recipes and other sources as required, checking ingredients with the home’s cooks and/or food service management.

RAs were trained to be as unobtrusive as possible. As care staff in the dining room was not aware of the specifics of data collection and only observed the RAs weighing residents’ food, we anticipate that care staff’s behaviours towards residents were unchanged. Multiple measures for mealtime scans and meal behaviour observations were averaged with standard deviations reviewed to determine any potential outliers.

There were relatively few challenges with all other data collection measures. Intensive in-person training of provincial coordinators and RAs was extremely important for ensuring quality of the data collection and data entry. All LTC homes submitted their survey and we surpassed care staff completion of the PDC questionnaire (goal: 320, *n* = 480 submitted), with three quarters being care staff with direct care responsibilities for residents (e.g., nursing). The small incentive was helpful in recruiting this number of care staff as was allowing staff to complete a paper form rather than relying solely on the REDCap™ form on the laptop. All DEAP and MTS were completed as per protocol, excepting two small dining lounges where only one MTS was completed rather than four. All research staff was in contact via a virtual internet group to promote communications. For example, RAs were encouraged to routinely communicate around nutrient analysis questions to promote consistency. Monthly teleconferences were held for the provincial lead investigators and provincial coordinators as a national group to share challenges and ensure consistency in data collection province-to-province. The provincial coordinator periodically reviewed the data collection at meals completed by the RAs. The primary investigator also routinely reviewed the data on REDcap™ to ensure consistency and accuracy.

A significant amount of time was spent by the lead provincial coordinator and a research analyst in cleaning and checking the data that was submitted on RedCAP™. Specifically, PG-SGA was checked by having the lead investigator review randomly selected participants from all provinces to ensure consistency in malnutrition classification based on the charted components of this measure, as this instrument does require clinical judgment. Some sites also found it challenging to report care staffing levels on the home survey. Checking was done in the data cleaning phase by confirming values with a home informant.

### Study feasibility

Pilot data were collected prior to the main study to confirm feasibility of all measures and the time necessary to complete the data collection in home. In each home, RAs were present for approximately 10–11 h per day for 15–20 days over a one-month period to collect residents’ food intake data. This was the most laborious and expensive portion of the data collection. They typically worked 3–4 days per week and had up to three, one-week breaks during the 1 year of data collection. Two provinces were able to complete data collection within ten months as it was the preference of the research staff to take fewer breaks. Two provinces did require overnight stays for research staff due to the commuting distance to the provincial research centre; the research budget accounted for these additional costs. RAs did have time between meals to conduct nutrient analysis of resident intake and menus; however, some ‘administrative’ days in each province were required when RAs were not in a LTC home to catch up on key aspects of data entry and cleaning. Cohesiveness of the provincial teams ensured data collection with minimal challenges, considering the scope and the breadth of this study.

## Summary

Poor food and fluid intake is a common but *preventable* problem in LTC. Inadequate intake leads to malnutrition, and with its high prevalence in LTC there is a subsequent significant impact on the quality of life, health, care, and costs for residents in LTC. The anticipated increase in need for LTC residences over the next three decades [83], as the baby-boomers age, necessitates solutions to the problem of poor food intake in LTC. Yet, effective solutions will continue to be elusive until this problem is fully described, including understanding the determinants of food intake. Multi-level data collection is needed to fully characterize the problem of inadequate food intake and this study will provide the necessary evidence to support improvements in policy and practice. The M3 study with its basis in an evidence-based conceptual framework, will address many of the current gaps in our understanding of this complex problem and provide a way forward to developing feasible and cost-effective solutions to poor food intake and malnutrition in LTC.

## Abbreviations

ADM: Alternative decision maker
DEAP: Dining Environment Assessment Protocol
DRI: Dietary reference intake
Ed-FED: Edinburgh Feeding Scale
ICC: Intra class correlation
LTC: Long term care
LTCF: Long term care facility
PG-SGA: Patient Generated- Subjective Global Assessment
M3: Making the Most Of Mealtimes
MDS: Minimum data set
MTS: Mealtime scan
RA: Research assistant
M-RCC: Mealime-Relational Care Checklist
STAND: Screening Tool for Acute Neuro Dysphagia
